# The effect of adding group-based counselling to individual lifestyle counselling on changes in dietary intake. The Inter99 study – a randomized controlled trial

**DOI:** 10.1186/1479-5868-5-59

**Published:** 2008-11-21

**Authors:** Ulla Toft, Lis Kristoffersen, Steen Ladelund, Lars Ovesen, Cathrine Lau, Charlotta Pisinger, Lisa von Huth Smith, Knut Borch-Johnsen, Torben Jørgensen

**Affiliations:** 1 Research Centre for Prevention and Health, Glostrup University Hospital, Building 84/85, DK-2600 Glostrup, Denmark; 2 Department of Gastroenterology, Slagelse Hospital, Ingemannsvej 18, DK-4200 Slagelse, Denmark; 3 Steno Diabetes Centre, Niels Steensensvej 2, DK-2820 Gentofte, Denmark

## Abstract

**Background:**

Few studies have investigated the specific effect of single intervention components in randomized controlled trials. The purpose was to investigate the effect of adding group-based diet and exercise counselling to individual life-style counselling on long-term changes in dietary habits.

**Methods:**

The study was a randomized controlled intervention study. From a general Danish population, aged 30 to 60 years (n = 61,301), two random sample were drawn (group A, n = 11,708; group B, n = 1,308). Subjects were invited for a health screening program. Participation rate was 52.5%. All participants received individual life-style counselling. Individuals at high risk of ischemic heart disease in group A were furthermore offered group-based life-style counselling. The intervention was repeated for high-risk individuals after one and three years. At five-year follow-up all participants were invited for a health examination. High risk individuals were included in this study (n = 2 356) and changes in dietary intake were analyzed using multilevel linear regression analyses.

**Results:**

At one-year follow-up group A had significantly increased the unsaturated/saturated fat ratio compared to group B and in men a significantly greater decrease in saturated fat intake was found in group A compared to group B (net change: -1.13 E%; P = 0.003). No differences were found between group A and B at three-year follow-up. At five-year follow-up group A had significantly increased the unsaturated/saturated fat ratio (net change: 0.09; P = 0.01) and the fish intake compared to group B (net change: 5.4 g/day; P = 0.05). Further, in men a non-significant tendency of a greater decrease was found at five year follow-up in group A compared to group B (net change: -0.68 E%; P = 0.10). The intake of fibre and vegetables increased in both groups, however, no significant difference was found between the groups. No differences between groups were found for saturated fat intake in women.

**Conclusion:**

Offering group-based counselling in addition to individual counselling resulted in small, but significantly improved dietary habits at five-year follow-up and a tendency of better maintenance, compared to individual counselling alone.

**Trial registration:**

The Inter99 study was approved by the local Ethics Committee (KA 98 155) and is registered with ClinicalTrials.gov (registration number: NCT00289237).

## Background

Nutrition is a major modifiable determinant of chronic disease, and there is increasing scientific evidence supporting that changes in diet have strong effects on health throughout life[1]. Thus in the primary prevention of chronic noncommunicable diseases in the population it is necessary to develop effective interventions to promote sustained improvements in dietary habits. Reviews of both Brunner et al[2] and Ammerman et al[3] found improved dietary habits of interventions which included dietary counselling, and Ebrahim & Davey Smith[4] found moderate but significant reductions in blood pressure, total cholesterol, weight and coronary risk score of individualized, non-pharmacological interventions on lifestyle. However, the majority of studies have been small, only involving highly selected individuals and been of relatively short duration lasting 12 months or less[2]. To attain and maintain healthy dietary habits is, however, a very intricate process, and research on adherence to lifestyle changes has shown an overall low compliance[5]. Multiple intervention strategies seem to be more effective in increasing compliance compared to single-strategy interventions [2,3,6,7] However, lifestyle intervention studies have in general been very heterogeneous and have tested multiple intervention components simultaneously, including dietary assessment, individual counselling, group sessions, social support, cooking lessons etc [2,6-9]. Whether all of the intervention strategies included in these complex interventions are necessary and which are the most effective is unclear.

The Inter99 study is a large randomised, non-pharmacological intervention study on lifestyle performed in a general population. The intervention lasted five years, focused on diet, physical activity and smoking habits and included health examination, individualised risk assessment of ischemic heart disease (IHD), repeated individual lifestyle counselling, and a group based diet and exercise course and/or a smoking cessation course were offered to high risk individuals. We have previously observed that this multi-factorial lifestyle intervention was effective in improving dietary habits in the intervention group (group A) compared with a non-intervention control group after five years[10]. The effect of an intensive lifestyle intervention on lifestyle and the incidence of IHD was the main focus of the study. But to be able to estimate the specific effect of offering participation at a group based diet and exercise course, a third smaller group (group B) was included in the study. The intervention in group B was similar to the intervention in group A but high risk individuals in group B were not offered group based counselling. Thus the aim of this paper was to investigate if there is an additional effect of offering a group based diet and exercise course to high risk individuals compared to a group only receiving health examination and individual lifestyle counselling.

## Methods

### Study population

Subjects were participants in the Inter99 study. The study investigated the effect of non-pharmacological multi-factorial lifestyle intervention on the incidence of IHD in a general population. The study has been described in detail elsewhere[11]. An age- and sex-stratified random sample of 13,016 individuals born in 1939–40, 1944–45, 1949–50, 1954–55, 1959–1960, 1964–65 and 1969–70 and living in 11 municipalities in the south-western part of Copenhagen County on 2^nd ^December 1998 was drawn from the Civil Registration by computer generated random numbers, and pre-randomised into two groups (group A – high intensity intervention, n = 11,708; group B – low intensity intervention; n = 1,308). Of the 13,016 people sampled, 82 were non-eligible, as they had died or could not be traced. The remaining 12,934 individuals were invited for a health-screening and lifestyle intervention program at The Research Centre for Prevention and Health. Invitation included a detailed questionnaire to be completed before attendance at the centre including information on socio-demographic and lifestyle factors. Written informed consent was obtained from all the participants. The Inter99 study was done in compliance with the Helsinki Declaration and was approved by the local Ethics Committee (KA 98 155) and is registered with ClinicalTrials.gov (registration number: NCT00289237).

Power calculations before study start were based on an expected participation rate of 70%. It was calculated that a difference in smoking reduction of 10% and a difference in reduction of cholesterol, systolic blood pressure and weight of 5% after 1 year between group A and B could be detected with an alpha = 0.05 and beta = 0.20.

A total of 6,906 persons in group A and B participated in the study. Of these, 122 subjects were excluded because of alcoholism, drug abuse or linguistic barriers leaving 6 784 (52.5%) for investigation.

### Physical examinations

All participants in the intervention group were required to be fasting from midnight on the day of attendance at the centre. Total-cholesterol was measured using a Refloctron^®^. Blood pressure was measured twice after 5 min of rest in lying position. Height was measured without shoes to the nearest 0.5 cm and weight measured without shoes and overcoat to the nearest 0.1 kg. A two hour oral glucose tolerance test (OGTT) was performed to diagnose impaired glucose tolerance (IGT) and diabetes[12].

For each participant the absolute risk of IHD within the next 10 years (the Copenhagen Risk Score) was estimated by entering information on age, sex, height, familial occurrence of acute myocardial infarction, previous IHD, diabetes, systolic blood pressure (lowest value), total cholesterol, weight and smoking into a computer program (PRECARD^®^)[13]. Individuals were categorised as high-risk individuals if they either had an absolute risk in the upper quintile of the distribution stratified according to sex and age or at least one of the following risk factors: systolic blood pressure ≥ 160 mmHg, total cholesterol ≥ 7.5 mmol/l, BMI ≥ 30 kg/m^2^, diabetes or IGT, or were daily smokers.

#### Intervention

Based on the personal risk estimate, each individual had a lifestyle counselling talk focussing on smoking, physical activity, diet and alcohol. The staff (doctors, nurses and dieticians) were all trained in health counselling and the motivational interviewing method[14]. The intervention was non-pharmacological. Participants with elevated levels of total cholesterol, blood pressure, plasma glucose or BMI in both group A and B were encouraged to contact their general practitioner. Participants in group A and B received the same individual lifestyle counselling.

In group A high risk individuals were in addition to the health screening program and the individual counselling offered group counselling on diet and physical activity or smoking cessation or reduction, depending on lifestyle and motivation to change lifestyle. The diet and exercise counselling groups were lead by a nurse or a dietician. Each group (15–20 individuals) was scheduled for six two-hour meetings during a four-six-months' period. The relatives of the participants were offered to participate in one of the meetings. More details on the intervention can be found at the website .

The overall goal was to achieve small but sustained dietary changes. More specifically the main emphasis was put on decreasing the total intake of saturated fat, substituting saturated fat for unsaturated fat, and increasing intake of fruits and vegetables, and fish.

#### Behavioural models implemented in the Inter99 intervention

Overall the intervention was based on elements from the Health Belief Model[15], the Social Cognitive Theory[16]and the Transtheoretical Model[17]. The implementation of these in the intervention is briefly described in the following.

A central educational tool in the Inter99 was the computerized program PRECARD^®^, a program based on the Health Belief Model[15]. The participants were given a thorough interpretation of the health examination results and the individual risk, using the PRECARD^® ^program, and were guided in how to make beneficial changes in health behaviour to improve their risk status. The individual lifestyle counselling was to a large degree based on The Transtheoretical Model which views behaviour change as a continuum involving several phases [17]. The staffs were taught how to identify at which stage the participant was, using specific questions from the questionnaire completed before the individual counselling, and from some initial questions in the counselling. Based on this the staffs sought to fit the counselling and advice given in agreement with the motivation of each participant. This was done based on a detailed guideline of which strategies to use and which messages to give at each stage in the model.

The Social Cognitive Theory[16] was primarily implemented in the group-based counselling by trying to promote higher self-efficacy through goal setting and modelling (using the group members as role models). In addition, it was implemented through focusing on the motivation underlying the actual behaviour and pointing out other behaviour options.

### Follow-up

All individuals in group A and B belonging to the high-risk group were re-invited after one and three years for a health examination, completion of questionnaires, a risk assessment and lifestyle counselling. Individuals who still fulfilled the criteria for being at high-risk in group A were again offered group counselling. Low-risk participants were followed by questionnaires. At five-year follow-up all participants at baseline were invited for health examination and a short finishing lifestyle counselling.

The present paper includes data for individuals identified as high risk individuals at baseline.

**Figure 1:**
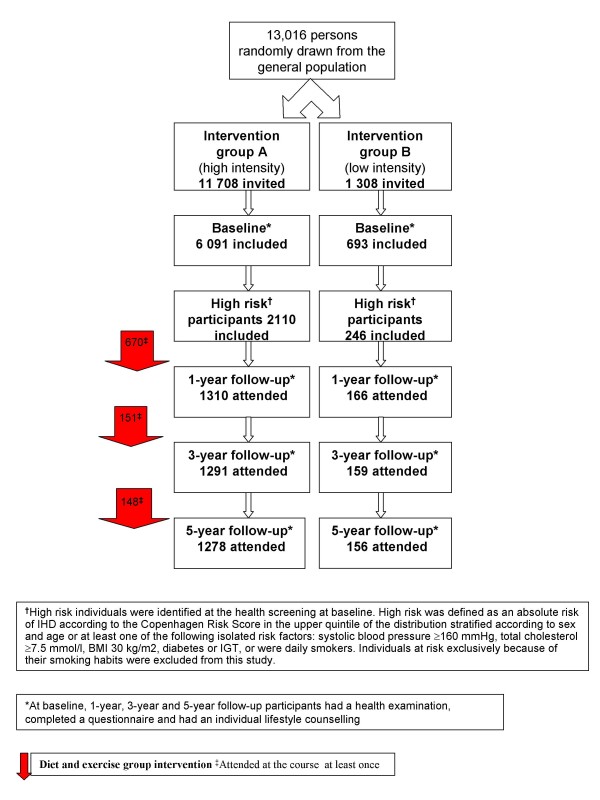
Flow-chart of the Inter99 study.

At baseline 60% (N = 3642) in group A and 59% (N = 411) in group B were categorised as high risk individuals. From these 25% were categorised as high risk individuals exclusively because they were daily smokers (A: N = 1508; B: N = 163) and these were not included in this paper. Dietary information were categorized as missing if no questions were answered in the FFQ on 5 or more pages out of 14 or the individual clearly had misunderstood the FFQ. Overall, 26 individuals (A: N = 24; B: N = 2) had missing information on dietary habits at all four occasions, leaving 2 356 (A: N = 2 110; B: N = 246) individuals for the analyses. Flowchart of the Inter99 study, including details on participant rates, is shown in figure 1. Figure 1 also shows the total number of individuals attending at least one diet and exercise group counselling session after baseline, one- and three-year follow-up (N = 670; 151; 148 respectively). If still at risk of IHD at one- and three years follow-up individuals in group A were repeatedly offered participation at the diet and exercise course. Overall 707 participated at one course, 116 at two and 10 individuals participated at three courses (data not shown in figure 1).

### Self-administered questionnaire

Education was defined on the basis of questions regarding number of years of vocational training categorized into four classes: 1: none, 2: ≤ 1 year, 3: 2–4 years, and 4: > 4 years. Employment status was classified as 1: employed and 2: has been employed/has never been employed. Living with a partner was defined as being married or cohabiting. Age was categorized in three groups (30–35; 40–50 and 55–60 years old). Smoking status was recorded in four categories: never smokers; ex-smokers; occasional smokers (< 1 g of tobacco per day); and daily smokers. To measure physical activity level in leisure time a previously developed and validated measure was used categorising participants into one of four categories: sedentary, moderate activity, regular exercise, and regular hard exercise[18]. Because of low numbers in the highest level of physical activity, the two highest classes were merged in the analyses. Self-rated health was reported as: "Excellent"; "Very good"; "Good"; "Fair"; and "Poor". Because of a low number of subjects answering excellent and poor, these categories were merged with very good and fair, respectively. Participants reported furthermore if they perceived that their dietary habits increased their risk of IHD: "Yes"; "No"; or "Do not know".

### Dietary intake

The participants completed a 198-item food frequency questionnaire (FFQ) during their visit at the centre. The FFQ is described and validated previously[19]. Briefly, participants were asked to report their average intake of different foods and beverages the last month, choosing between seven and eleven possible responses, ranging from never to eight or more times a day. The questionnaire also included questions about the types of bread, spread and fat used for cooking. The food consumption quantity was obtained by multiplying the frequency of consumption of each unit of food by standard portion sizes[20]. To translate food consumption into energy intake and daily nutrient intake all food items were linked to food items in the Danish Food Composition Databank version 6[21]. The software program FoodCalc version 1.3 was used for the calculations[22].

### Statistical analyses

Statistical analyses were made including all high risk individuals in group A and B (intention-to-treat) who reported dietary habits in at least one of the four occasions (baseline, one year, three year and five year follow-up). To investigate differences between groups of dietary intake during the five years of intervention, multilevel regression analyses with repeated measurements were made. Data were analysed using Proc Mixed in SAS (SAS statistical software, version 9.1, SAS Institute Inc., Cary, NC, USA) with normally distributed random intercepts. The statistical analyses were done for the nutritional factors focused on in the dietary intervention (fat, especially unsaturated-saturated fat ratio, fibre, fish, fruit and vegetables). To minimize bias due to loss to follow-up covariates were included in the multi-adjusted analyses if they were differently distributed at baseline between participants and dropouts at one, three and five-year follow-up. Furthermore, adjustments were made for covariates differently distributed between group A and B at baseline.

Drop-out was defined as missing information on food intake at follow-up, either because the participant did not attend or because he did not complete the questionnaire. Multiple logistic regression analysis was used to compare participants and drop-outs at one, three and five year follow-up, respectively. Additionally, all multi-adjusted analyses were made with and without adjustment for energy intake and when analysing the development in the ratio between unsaturated and saturated fat adjustments were made for total fat intake.

To investigate whether the associations differed between men and women, interaction terms between sex, time and group were included in the models and if significant, analyses were stratified by sex. A p-value of 5% was considered significant.

## Results

### Baseline comparisons

No significant differences were found in baseline characteristics between the two intervention groups (table 1). Similarly no differences were found in baseline food intake between the two groups except for a significantly higher intake of fruit in men in group B (table 2).

**Table 1:** Baseline characteristics according to sex and intervention group in 2356 subjects from the Inter99 study.

	**Men (%)**		**Women (%)**	
	
	Group A N = 1048	Group B N = 132	Group A N = 1062	Group B N = 114
**Age **(years)^*a*^				
30–35	7	9	13	11
40–50	59	56	55	60
55–60	34	35	32	29

**Vocational training**^*a*^				
None	16	15	22	23
< 2 years	3	3	7	7
2 ≤ years ≤ 4	65	65	64	66
4 < years ≤ 9	16	17	7	4

**Employed**^*a*^				
Yes	87	90	79	82

**Living with partner**^*a*^				
Yes	84	88	80	81

**Daily smoking**^*a*^				
Yes	34	33	28	30

**Alcohol **(units alcohol/week)^*a*^				
0	7	6	19	13
1–7	30	36	52	53
8–14	22	16	18	20
14–21	15	13	6	6
21+	26	29	5	8

**Physical activity**^*a*^				
Mainly sedentary	27	23	26	28
Moderate activity	58	61	65	62
Regular/heavy exercise	15	16	9	10

**Perceived risk of diet**^*a*^				
Yes	20	15	15	15

**Self-rated health**^*a*^				
Excellent/very good	26	32	21	26
Good	61	54	61	60
Fair/poor	13	14	18	14

	**Mean (sd)**			

**BMI**^*b*^	29.7(4.6)	29.1(4.1)	29.9(6.0)*	30.0(6.0)
**Total plasma cholesterol**^*b*^	5.9(1.2)	5.8(1.3)	5.8(1.1)*	5.6(1.6)
**Systolic blood pressure**^*b*^	144(18)*	140(20)	138(20)*	133(18)

Differences between groups were tested by: ^*a *^Chi^2^-test.

^*b *^Wilcoxon two sample test. *Difference between groups, P < 0.05

**Table 2:** Crude dietary intake at baseline

	**Men Median (P5, P95)^a^**		**Women Median (P5, P95)^a^**	
	Group A N = 1048	Group B N = 132	Group A N = 1062	Group B N = 114

**Energy (MJ)**	10.0 (5.9–17.7)	10.3 (6.0–16.7)	8.3 (4.4–14.8)	7.9 (5.0–14.3)
**Total fat (% of energy)**	33.7 (21.9–45.0)	33.3 (22.0–44.1)	30.7 (19.6–42.2)	29.2 (19.4–43.0)
**Saturated fat (% of energy)**	12.4 (7.2–18.4)	12.4 (7.0–18.5)	11.3 (6.2–17.4)	11.1 (6.2–17.0)
**Polyunsaturated fat (% of energy)**	5.2 (3.3–8.4)	5.3 (3.3–8.3)	5.0 (3.1–7.8)	4.8 (3.1–7.7)
**Monounsaturated fat (% of energy)**	10.9 (6.6–15.2)	10.9 (5.7–14.5))	9.7 (5.8–14.0)	9.6 (5.9–15.0)
**Carbohydrate (% of energy)**	44.3 (33.2–57.5)	44.2 (34.7–57.6)	50.0 (36.8–63.4)	49.7 (38.1–61.8)
**Protein (% of energy)**	15.0 (11.3–20.1)	14.8 (11.0–19.3)	15.2 (11.4–20.3)	15.3 (11.3–20.0))
**Fruits (g/day)**	74.6* (4.2–439)	83.9 (10.4–489)	149 (11.5–580)	122.6 (13.4–607)
**Vegetables (g/day)**	90.1 (22.2–251)	91.0 (29.8–198)	111.6 (26.6–368)	120.9 (24.9–332)
**Fish (g/day)**	27.8 (0.9–102.9)	24.4 (0–116.8)	20.7 (0–74.0)	19.9 (0–93.2)

^a^P5 = 5^th ^percentile; P95 = 95^th ^percentile. Comparisons of differences in intake between groups were done by Wilcoxon two sample test. *Difference between groups, P < 0.05

Baseline dietary intake (energy, macronutrients, fruits, vegetables and fish) did not differ between responders and non-responders, except in female participants in group A, those who did not respond at five-year follow-up, had a higher intake of saturated fat. Compared with responders, non-responders at one-, three-, and five-year follow-up were at baseline in general more likely to be younger, daily smokers, unemployed, and had no or maximum one year of vocational training. Furthermore, they had low self-rated health, high intake of alcohol and perceived their dietary habits as a health risk. More details on differences between groups at baseline and between responders and non-responders at baseline have been described elsewhere[11].

### Follow-up data

The results from the multilevel regression analyses comparing dietary changes in group A and B are described in the following. The main focus in the interpretation of the intervention effect was on differences in the *development *in the dietary intake during the five years of intervention.

**Figure 2:**
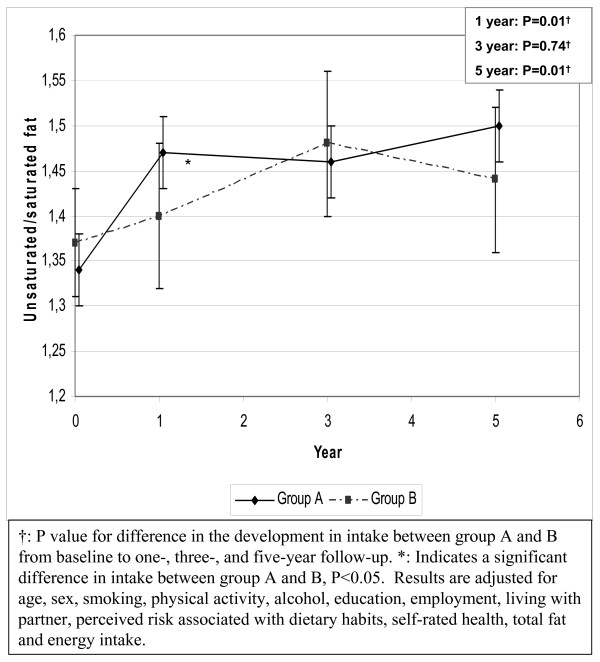
Development in the unsaturated-saturated fat ratio from baseline to five-year follow-up.

The unsaturated/saturated fat ratio increased in both groups during the five years of intervention (figure 2). Group A had increased the ratio significantly more than group B at both one and five year follow-up, adjusted for total fat and energy intake and multiple confounders (net change at five-year follow-up: 0.09; P = 0.01).

**Figure 3:**
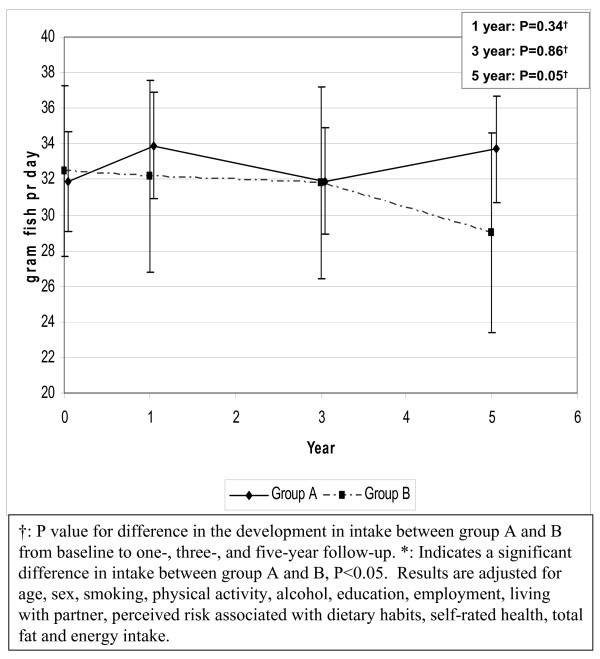
Development in the intake of fish from baseline to five year follow-up.

A small increase in group A and a small decrease in group B were found in the intake of fish (figure 3). The development was significantly different between groups at five year follow-up (net change: 5.4 g/day; P = 0.05). Similar results were found after further adjustment for energy intake (data not shown).

The intake of fibre and vegetables increased in both groups, however, no significant difference was found between the groups (data not shown). Similar the energy intake decreased in both intervention groups (group A: men: -774 kJ/day; women:-882 kJ/day; group B: men: -1330 kJ/day; women -890 kJ/day) but no significant differences between groups were found.

**Figure 4:**
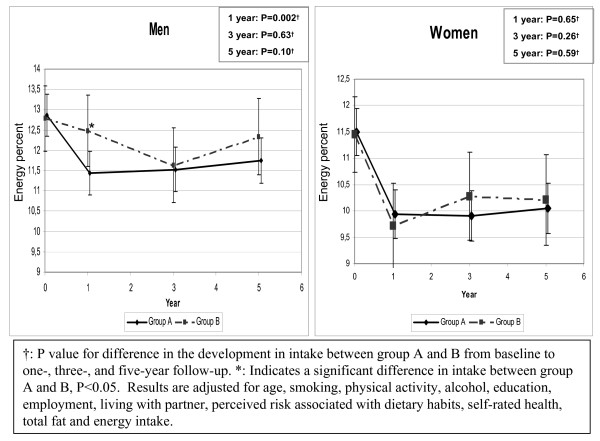
Development in the intake of saturated fat (E%) from baseline to five year follow-up.

Significant interaction terms with sex were found for saturated fat and fruit intake and therefore these analyses were made for men and women separately. The fruit intake increased in both groups. In men no significant differences were found between groups. In women at one year follow-up the increase was significantly lower in group A (net change: -50 g/day; P = 0.03) whereas at three- and five year follow-up no significant differences between groups were found (data not shown). Saturated fat decreased in both groups (figure 4). In men a significantly greater decrease was found in group A compared to group B at 1 year follow-up (net change: -1.13 E%; P = 0.003) and a non-significant tendency of a greater decrease in group A was found at five year follow-up (net change: -0.68 E%; P = 0.10). In women no differences in saturated fat intake were found between group A and B.

Adjustment for energy intake did show different results when comparing development in dietary intake between group A and B.

## Discussion

Adding group-based diet and exercise counselling to an intervention including individual risk assessment and individual lifestyle counselling resulted in a significant, albeit small, additional improvement in the unsaturated/saturated fat ratio and fish intake. No significant effect of adding group-based counselling was found for fruit, vegetables and fibre intake. Overall, the results showed a tendency of an equal effect in group A and B at three years follow-up whereas at five years follow-up there was a tendency that group B to a lesser degree maintained the beneficial dietary changes.

Few studies have investigated the specific effect of single intervention components in multi-factorial studies however, to our knowledge this is the first large-scale, long-term randomised lifestyle intervention performed in the general population, investigating the additional effect on food intake of offering group counselling to high risk individuals. One of the important early researchers in the field of the effect of group interventions on health behaviour was the social psychologist Kurt Lewin. He found a significantly greater effect on changes in dietary habits in women who joined a group compared to those receiving individual counselling[23]. Recently, a Cochrane review by Deakin et al[24] found that group-based training of diabetes patients was more effective in reducing body weight, blood pressure, fasting blood glucose and glucated haemoglobin compared with routine care delivered on an individual basis. Another Cochrane review of the effect of group-based training on smoking habits found no evidence that group therapy was more effective than individual counselling[25].

In our study the effect of additionally offering group-based counselling on diet and exercise was found to be small, indicating that the cost-effectiveness of offering group-based counselling in addition to repeated individualised risk assessment and lifestyle counselling might be low. However, the results are based on analyses including all high risk individuals in the two groups, whereas only 47% of the individuals in group A actually joined the diet and exercise group course. Analyses performed separately for participants attending at least once at the diet and exercise course showed more pronounced effects of group counselling (data not shown). Furthermore, the low participation rate in general might be an important explanation for the few significant differences between the two intervention groups. The expected participation rate of the Inter99 was 70%, whereas the actual rate was 52.5%. Thus the assumptions behind the power calculation of the required sample size of group B were not fulfilled; hence the power to detect the effect of offering group counselling was considerably smaller than expected.

Thus, the low participation rate at the group-based counselling might be an important reason for the small effect on dietary habits found. To improve participation rate it is relevant to identify mediators of participation and adherence to the course. This was done in an earlier study. We found that important mediators of participation at the diet and exercise was awareness of unhealthy lifestyle, perceived susceptibility of disease, motivation towards lifestyle changes, recent diagnosis of IGT/diabetes and overweight[26]. To improve participation rate in future interventions it is therefore relevant to focus on facilitation these mediators in the individual counselling. However, it could be argued that not all individuals might benefit from participating in the group-based course. Therefore, it is relevant to identify characteristics of those who actually benefit from participating and in future interventions to offer and tailor the group-based intervention to these individuals.

To minimize the bias due to the low participant rate and the high degree of loss to follow-up when investigating the effect of the intervention we used hierarchical multilevel regression analyses with repeated measurements and random effects. These analyses make it possible to take into account the loss to follow-up under the assumption of missing at random (MAR)[27] by including covariates associated with missing information on dietary habits at follow-ups. The method is superior to two often-used methods: "last observation carried forward" and "complete case analysis". These methods are based on the assumption of missing completely at random (MCAR) which implicitly means the excluded/lost individuals do not differ systematically from the included individuals[28]. Under the assumption of MAR, missing values and dropouts are allowed to depend on previously observed outcomes. Thus, because baseline intake and other relevant characteristics were taken into account in the model the fact that these to some degree varied between responders and non-responders at follow-ups should therefore not bias the results using this method.

The validation of the FFQ showed as earlier described that the questionnaire provided a reasonable quantitative assessment of dietary intake and a good classification of individuals. However, the results for saturated fat showed a tendency of increased underreporting according to the FFQ with increasing intake of saturated fat. In line with this the degree of underreporting seems to be highest in men who in general have a higher intake of saturated fat. This could potentially have attenuated some of the intervention effect. The validation of the dietary intake was made for "point-in-time" associations only. However, there is substantial evidence that change in diet is hard to estimate precisely[29]. Thus, in this study it would have been relevant to investigate if the FFQ was sufficiently sensitive for measuring dietary changes but for economical reasons this was not done. Overall, misclassification due to difficulties in measuring dietary changes would tend to lead to underestimation of the intervention effect.

The net change in fish intake was 5.4 g/day (in women 8.5 g/day, data not shown). He et al (2004) found a 7% lower risk of CHD mortality for each 20 g/day increase in fish intake for all participants included in the meta-analysis. The net-changes in men in the saturated fat intake were in this study -1.13 E% at one year follow-up and -0.68 E% at five year follow-up. Osler et al[30] estimated that a reduction of 1 E% in saturated fat intake would reduce the risk of heart disease in the population with 3%. Further, when aggregated across the entire dietary pattern, several small changes in food habits may lead to greater health gains than the above estimates would suggest[31]. Thus, the small, but long-term changes in dietary habits, if sustained, might prove to have a significant effect on the prevention of IHD. The exact effect on morbidity and mortality in the population of these dietary changes is however, impossible to estimate at this point of time. Fortunately, in Denmark it is possible to follow the whole study population in central registers, including the National Hospital Registry and the Cause of Death Registry. From these it will, in the coming years be possible to estimate the effect of the intervention on the incidence of e.g. IHD.

The strategy in the Inter99 study is categorized as a high-risk strategy as it was based on identifying high-risk individuals and targeting the intervention to the risk profile of the individual. However, the intervention was not limited to high-risk individuals but was performed in an unselected general population. Thus, although the intervention differentiated the intensity of the intervention according to the risk profile and lifestyle of the individuals, both high and low-risk individuals had a thorough health examination and individual lifestyle counselling. Thus, the intervention could potentially shift the distribution of lifestyle and risk factors of the whole population of participants. It could thereby promote significant improvement in the health of the population as a whole.

To decrease dropout rates it is important to explore important mediators for participation and adherence throughout the study. However, this will not improve the participation rate. Several reviews have focused on the attitudes and characteristics of individuals unwilling or unable to participate in randomized controlled intervention studies [32-34]. These attitudes and characteristics differ in many ways from the participants; including participants being in general older, married and having a higher socioeconomic status and education level than non-participants[32]. This means both a lower external validity of the studies but also that individual intervention studies performed in the general population are likely to increase the social inequality in health. To reach those not willing to participate in for example preventive lifestyle programs it is necessary to use other strategies than the high-risk strategy. For this, a more population-based strategy should be prioritized in combination with the high-risk strategy.

## Conclusion

Offering group-based diet and exercise counselling in addition to individual lifestyle counselling resulted in significant positive changes in fish intake and the ratio between unsaturated and saturated fat compared to individual lifestyle counselling only. No significant effect was found on the intake of fruit, vegetables and fibre. There was a tendency that group-based counselling improved maintenance of positive dietary changes.

## Competing interests

The authors declare that they have no competing interests.

## Authors' contributions

TJ was responsible for the design and conduct of the Inter99 study. UT was responsible for conducting the data analysis, interpretation of the data and writing of the manuscript. All the authors participated in editing the manuscript and provided advice regarding interpretation of the results. None of the contributing authors had any financial or personal interests in any of the bodies sponsoring this research. All authors read and approved the final manuscript.
